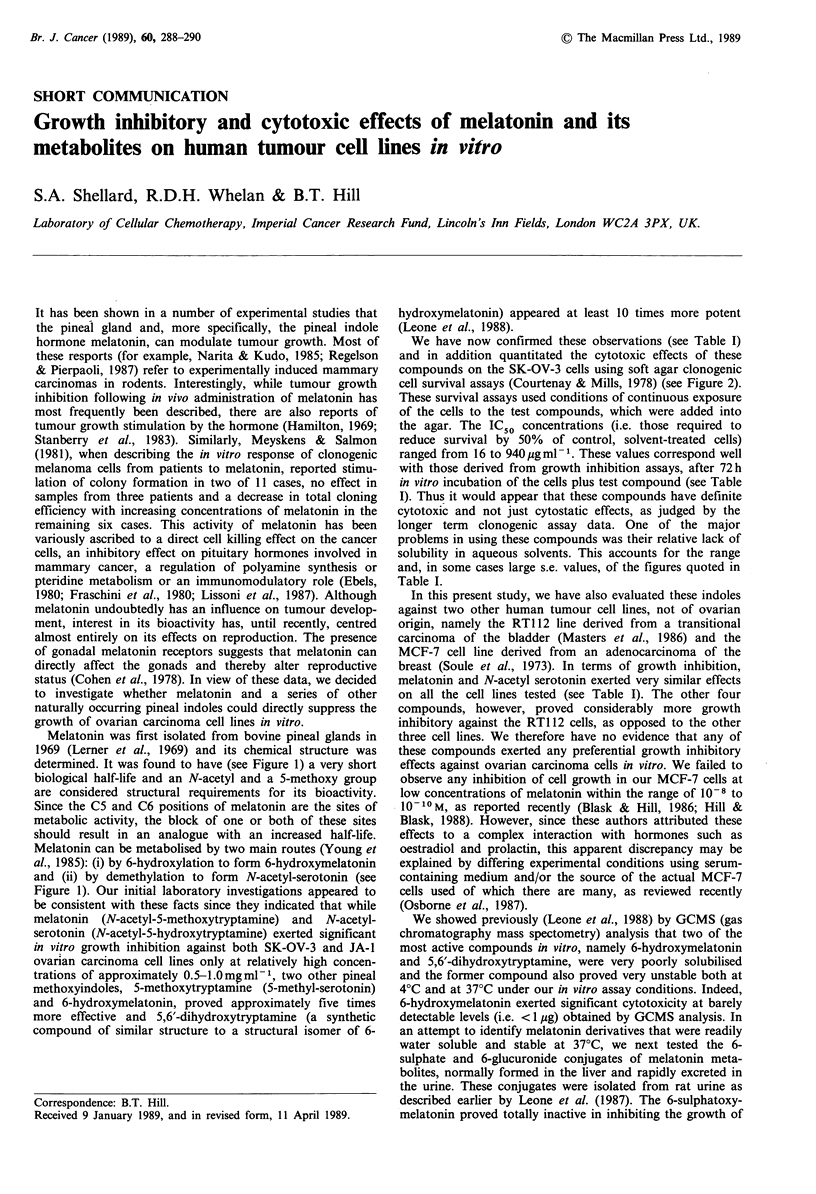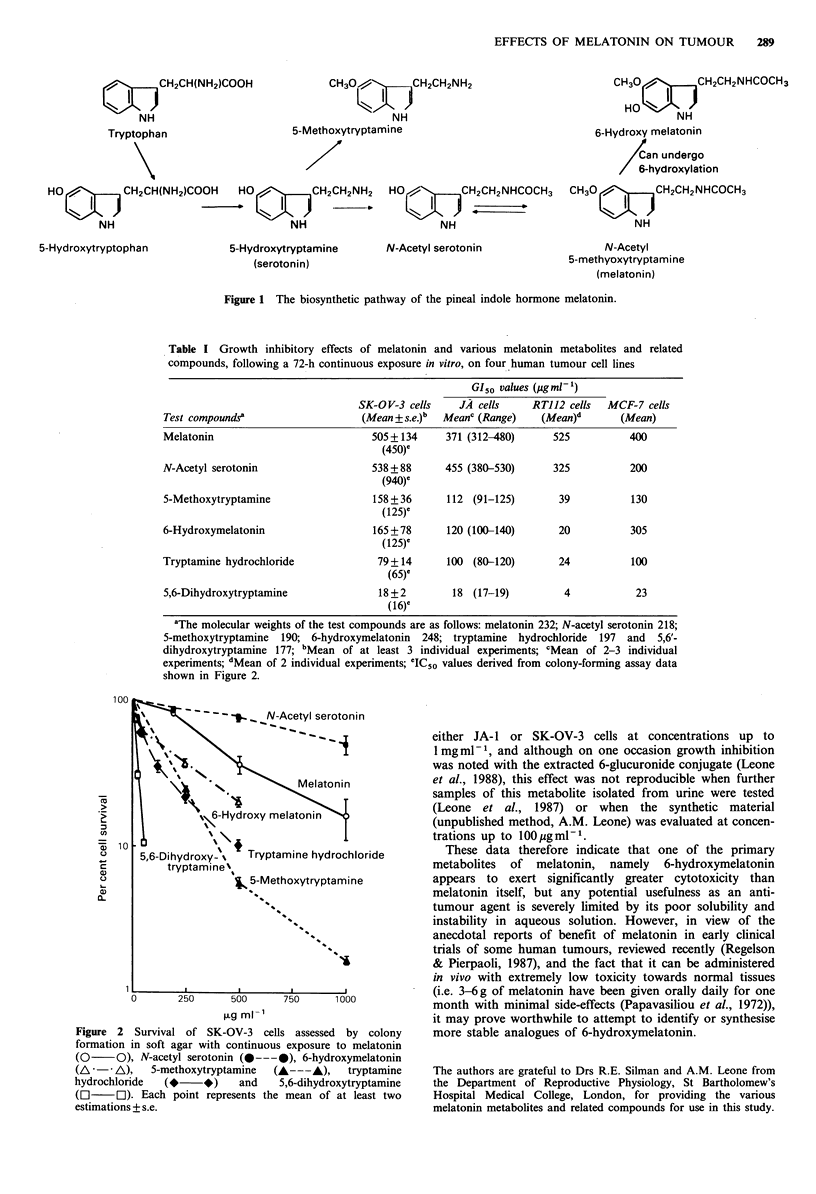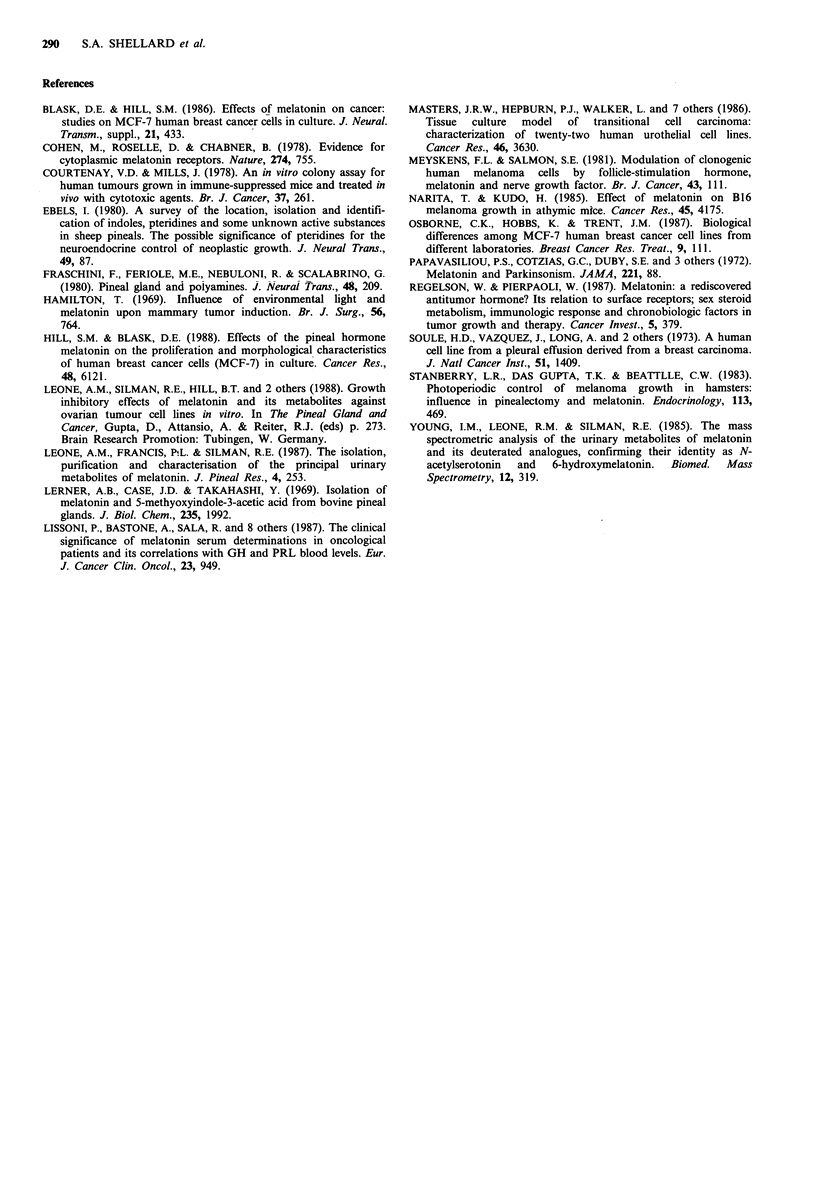# Growth inhibitory and cytotoxic effects of melatonin and its metabolites on human tumour cell lines in vitro.

**DOI:** 10.1038/bjc.1989.272

**Published:** 1989-09

**Authors:** S. A. Shellard, R. D. Whelan, B. T. Hill

**Affiliations:** Laboratory of Cellular Chemotherapy, Imperial Cancer Research Fund, Lincoln's Inn Fields, London, UK.


Br. J. Cancer (1989), 60, 288-290 © The Macmillan Press Ltd., 1989

SHORT COMMUNICATION

### Growth inhibitory and cytotoxic effects of melatonin and its metabolites on human tumour cell lines *in vitro*

S.A. Shellard, R.D.H. Whelan & B.T. Hill

*Laboratory of Cellular Chemotherapy, Imperial Cancer Research Fund, Lincoln's Inn Fields, London WC2A 3PX, UK.*

It has been shown in a number of experimental studies that the pineal gland and, more specifically, the pineal indole hormone melatonin, can modulate tumour growth. Most of these resports (for example, Narita & Kudo, 1985; Regelson & Pierpaoli, 1987) refer to experimentally induced mammary carcinomas in rodents. Interestingly, while tumour growth inhibition following *in vivo* administration of melatonin has most frequently been described, there are also reports of tumour growth stimulation by the hormone (Hamilton, 1969; Stanberry *et al.*, 1983). Similarly, Meyskens & Salmon (1981), when describing the *in vitro* response of clonogenic melanoma cells from patients to melatonin, reported stimulation of colony formation in two of 11 cases, no effect in samples from three patients and a decrease in total cloning efficiency with increasing concentrations of melatonin in the remaining six cases. This activity of melatonin has been variously ascribed to a direct cell killing effect on the cancer cells, an inhibitory effect on pituitary hormones involved in mammary cancer, a regulation of polyamine synthesis or pteridine metabolism or an immunomodulatory role (Ebels, 1980; Fraschini *et al.*, 1980; Lissoni *et al.*, 1987). Although melatonin undoubtedly has an influence on tumour development, interest in its bioactivity has, until recently, centred almost entirely on its effects on reproduction. The presence of gonadal melatonin receptors suggests that melatonin can directly affect the gonads and thereby alter reproductive status (Cohen *et al.*, 1978). In view of these data, we decided to investigate whether melatonin and a series of other naturally occurring pineal indoles could directly suppress the growth of ovarian carcinoma cell lines *in vitro*.

Melatonin was first isolated from bovine pineal glands in 1969 (Lerner *et al.*, 1969) and its chemical structure was determined. It was found to have (see Figure 1) a very short biological half-life and an *N*-acetyl and a 5-methoxy group are considered structural requirements for its bioactivity. Since the C5 and C6 positions of melatonin are the sites of metabolic activity, the block of one or both of these sites should result in an analogue with an increased half-life. Melatonin can be metabolised by two main routes (Young *et al.*, 1985): (i) by 6-hydroxylation to form 6-hydroxymelatonin and (ii) by demethylation to form *N*-acetyl-serotonin (see Figure 1). Our initial laboratory investigations appeared to be consistent with these facts since they indicated that while melatonin (*N*-acetyl-5-methoxytryptamine) and *N*-acetyl-serotonin (*N*-acetyl-5-hydroxytryptamine) exerted significant *in vitro* growth inhibition against both SK-OV-3 and JA-1 ovarian carcinoma cell lines only at relatively high concentrations of approximately 0.5–1.0 mg ml$^{-1}$, two other pineal methoxyindoles, 5-methoxytryptamine (5-methyl-serotonin) and 6-hydroxymelatonin, proved approximately five times more effective and 5,6′-dihydroxytryptamine (a synthetic compound of similar structure to a structural isomer of 6-hydroxymelatonin) appeared at least 10 times more potent (Leone *et al.*, 1988).

We have now confirmed these observations (see Table I) and in addition quantitated the cytotoxic effects of these compounds on the SK-OV-3 cells using soft agar clonogenic cell survival assays (Courtenay & Mills, 1978) (see Figure 2). These survival assays used conditions of continuous exposure of the cells to the test compounds, which were added into the agar. The $IC_{50}$ concentrations (i.e. those required to reduce survival by 50% of control, solvent-treated cells) ranged from 16 to 940 μg ml$^{-1}$. These values correspond well with those derived from growth inhibition assays, after 72 h *in vitro* incubation of the cells plus test compound (see Table I). Thus it would appear that these compounds have definite cytotoxic and not just cytostatic effects, as judged by the longer term clonogenic assay data. One of the major problems in using these compounds was their relative lack of solubility in aqueous solvents. This accounts for the range and, in some cases large s.e. values, of the figures quoted in Table I.

In this present study, we have also evaluated these indoles against two other human tumour cell lines, not of ovarian origin, namely the RT112 line derived from a transitional carcinoma of the bladder (Masters *et al.*, 1986) and the MCF-7 cell line derived from an adenocarcinoma of the breast (Soule *et al.*, 1973). In terms of growth inhibition, melatonin and *N*-acetyl serotonin exerted very similar effects on all the cell lines tested (see Table I). The other four compounds, however, proved considerably more growth inhibitory against the RT112 cells, as opposed to the other three cell lines. We therefore have no evidence that any of these compounds exerted any preferential growth inhibitory effects against ovarian carcinoma cells *in vitro*. We failed to observe any inhibition of cell growth in our MCF-7 cells at low concentrations of melatonin within the range of $10^{-8}$ to $10^{-10}$ M, as reported recently (Blask & Hill, 1986; Hill & Blask, 1988). However, since these authors attributed these effects to a complex interaction with hormones such as oestradiol and prolactin, this apparent discrepancy may be explained by differing experimental conditions using serum-containing medium and/or the source of the actual MCF-7 cells used of which there are many, as reviewed recently (Osborne *et al.*, 1987).

We showed previously (Leone *et al.*, 1988) by GCMS (gas chromatography mass spectometry) analysis that two of the most active compounds *in vitro*, namely 6-hydroxymelatonin and 5,6′-dihydroxytryptamine, were very poorly solubilised and the former compound also proved very unstable both at 4°C and at 37°C under our *in vitro* assay conditions. Indeed, 6-hydroxymelatonin exerted significant cytotoxicity at barely detectable levels (i.e. $<1$ μg) obtained by GCMS analysis. In an attempt to identify melatonin derivatives that were readily water soluble and stable at 37°C, we next tested the 6-sulphate and 6-glucuronide conjugates of melatonin metabolites, normally formed in the liver and rapidly excreted in the urine. These conjugates were isolated from rat urine as described earlier by Leone *et al.* (1987). The 6-sulphatoxy-melatonin proved totally inactive in inhibiting the growth of

Correspondence: B.T. Hill.
Received 9 January 1989, and in revised form, 11 April 1989.

**Figure 1** The biosynthetic pathway of the pineal indole hormone melatonin.

**Table I** Growth inhibitory effects of melatonin and various melatonin metabolites and related compounds, following a 72-h continuous exposure *in vitro*, on four human tumour cell lines

| | $GI_{50}$ values ($\mu g\,ml^{-1}$) | | | |
|---|---|---|---|---|
| *Test compounds*[a] | *SK-OV-3 cells (Mean ± s.e.)*[b] | *JA cells Mean*[c] *(Range)* | *RT112 cells (Mean)*[d] | *MCF-7 cells (Mean)* |
| Melatonin | 505±134 (450)[e] | 371 (312–480) | 525 | 400 |
| *N*-Acetyl serotonin | 538±88 (940)[e] | 455 (380–530) | 325 | 200 |
| 5-Methoxytryptamine | 158±36 (125)[e] | 112 (91–125) | 39 | 130 |
| 6-Hydroxymelatonin | 165±78 (125)[e] | 120 (100–140) | 20 | 305 |
| Tryptamine hydrochloride | 79±14 (65)[e] | 100 (80–120) | 24 | 100 |
| 5,6-Dihydroxytryptamine | 18±2 (16)[e] | 18 (17–19) | 4 | 23 |

[a]The molecular weights of the test compounds are as follows: melatonin 232; *N*-acetyl serotonin 218; 5-methoxytryptamine 190; 6-hydroxymelatonin 248; tryptamine hydrochloride 197 and 5,6'-dihydroxytryptamine 177; [b]Mean of at least 3 individual experiments; [c]Mean of 2–3 individual experiments; [d]Mean of 2 individual experiments; [e]$IC_{50}$ values derived from colony-forming assay data shown in Figure 2.

**Figure 2** Survival of SK-OV-3 cells assessed by colony formation in soft agar with continuous exposure to melatonin (○——○), *N*-acetyl serotonin (●---●), 6-hydroxymelatonin (△·—·△), 5-methoxytryptamine (▲---▲), tryptamine hydrochloride (◆——◆) and 5,6-dihydroxytryptamine (□——□). Each point represents the mean of at least two estimations ± s.e.

either JA-1 or SK-OV-3 cells at concentrations up to 1 mg ml$^{-1}$, and although on one occasion growth inhibition was noted with the extracted 6-glucuronide conjugate (Leone *et al.*, 1988), this effect was not reproducible when further samples of this metabolite isolated from urine were tested (Leone *et al.*, 1987) or when the synthetic material (unpublished method, A.M. Leone) was evaluated at concentrations up to 100 $\mu$g ml$^{-1}$.

These data therefore indicate that one of the primary metabolites of melatonin, namely 6-hydroxymelatonin appears to exert significantly greater cytotoxicity than melatonin itself, but any potential usefulness as an anti-tumour agent is severely limited by its poor solubility and instability in aqueous solution. However, in view of the anecdotal reports of benefit of melatonin in early clinical trials of some human tumours, reviewed recently (Regelson & Pierpaoli, 1987), and the fact that it can be administered *in vivo* with extremely low toxicity towards normal tissues (i.e. 3–6 g of melatonin have been given orally daily for one month with minimal side-effects (Papavasiliou *et al.*, 1972)), it may prove worthwhile to attempt to identify or synthesise more stable analogues of 6-hydroxymelatonin.

The authors are grateful to Drs R.E. Silman and A.M. Leone from the Department of Reproductive Physiology, St Bartholomew's Hospital Medical College, London, for providing the various melatonin metabolites and related compounds for use in this study.

### References

BLASK, D.E. & HILL, S.M. (1986). Effects of melatonin on cancer: studies on MCF-7 human breast cancer cells in culture. *J. Neural. Transm.*, suppl., **21**, 433.

COHEN, M., ROSELLE, D. & CHABNER, B. (1978). Evidence for cytoplasmic melatonin receptors. *Nature*, **274**, 755.

COURTENAY, V.D. & MILLS, J. (1978). An *in vitro* colony assay for human tumours grown in immune-suppressed mice and treated *in vivo* with cytotoxic agents. *Br. J. Cancer*, **37**, 261.

EBELS, I. (1980). A survey of the location, isolation and identification of indoles, pteridines and some unknown active substances in sheep pineals. The possible significance of pteridines for the neuroendocrine control of neoplastic growth. *J. Neural Trans.*, **49**, 87.

FRASCHINI, F., FERIOLE, M.E., NEBULONI, R. & SCALABRINO, G. (1980). Pineal gland and polyamines. *J. Neural Trans.*, **48**, 209.

HAMILTON, T. (1969). Influence of environmental light and melatonin upon mammary tumor induction. *Br. J. Surg.*, **56**, 764.

HILL, S.M. & BLASK, D.E. (1988). Effects of the pineal hormone melatonin on the proliferation and morphological characteristics of human breast cancer cells (MCF-7) in culture. *Cancer Res.*, **48**, 6121.

LEONE, A.M., SILMAN, R.E., HILL, B.T. and 2 others (1988). Growth inhibitory effects of melatonin and its metabolites against ovarian tumour cell lines *in vitro*. In *The Pineal Gland and Cancer,* Gupta, D., Attansio, A. & Reiter, R.J. (eds) p. 273. Brain Research Promotion: Tubingen, W. Germany.

LEONE, A.M., FRANCIS, P.L. & SILMAN, R.E. (1987). The isolation, purification and characterisation of the principal urinary metabolites of melatonin. *J. Pineal Res.*, **4**, 253.

LERNER, A.B., CASE, J.D. & TAKAHASHI, Y. (1969). Isolation of melatonin and 5-methyoxyindole-3-acetic acid from bovine pineal glands. *J. Biol. Chem.*, **235**, 1992.

LISSONI, P., BASTONE, A., SALA, R. and 8 others (1987). The clinical significance of melatonin serum determinations in oncological patients and its correlations with GH and PRL blood levels. *Eur. J. Cancer Clin. Oncol.*, **23**, 949.

MASTERS, J.R.W., HEPBURN, P.J., WALKER, L. and 7 others (1986). Tissue culture model of transitional cell carcinoma: characterization of twenty-two human urothelial cell lines. *Cancer Res.*, **46**, 3630.

MEYSKENS, F.L. & SALMON, S.E. (1981). Modulation of clonogenic human melanoma cells by follicle-stimulation hormone, melatonin and nerve growth factor. *Br. J. Cancer*, **43**, 111.

NARITA, T. & KUDO, H. (1985). Effect of melatonin on B16 melanoma growth in athymic mice. *Cancer Res.*, **45**, 4175.

OSBORNE, C.K., HOBBS, K. & TRENT, J.M. (1987). Biological differences among MCF-7 human breast cancer cell lines from different laboratories. *Breast Cancer Res. Treat.*, **9**, 111.

PAPAVASILIOU, P.S., COTZIAS, G.C., DUBY, S.E. and 3 others (1972). Melatonin and Parkinsonism. *JAMA*, **221**, 88.

REGELSON, W. & PIERPAOLI, W. (1987). Melatonin: a rediscovered antitumor hormone? Its relation to surface receptors; sex steroid metabolism, immunologic response and chronobiologic factors in tumor growth and therapy. *Cancer Invest.*, **5**, 379.

SOULE, H.D., VAZQUEZ, J., LONG, A. and 2 others (1973). A human cell line from a pleural effusion derived from a breast carcinoma. *J. Natl Cancer Inst.*, **51**, 1409.

STANBERRY, L.R., DAS GUPTA, T.K. & BEATTLLE, C.W. (1983). Photoperiodic control of melanoma growth in hamsters: influence in pinealectomy and melatonin. *Endocrinology*, **113**, 469.

YOUNG, I.M., LEONE, R.M. & SILMAN, R.E. (1985). The mass spectrometric analysis of the urinary metabolites of melatonin and its deuterated analogues, confirming their identity as *N*-acetylserotonin and 6-hydroxymelatonin. *Biomed. Mass Spectrometry*, **12**, 319.